# Severity of dental caries and risk of coronary heart disease in middle-aged men and women: a population-based cohort study of Korean adults, 2002–2013

**DOI:** 10.1038/s41598-019-47029-3

**Published:** 2019-07-19

**Authors:** Kyuwoong Kim, Seulggie Choi, Jooyoung Chang, Sung Min Kim, Seon Jip Kim, Ryan Jin-Young Kim, Hyun-Jae Cho, Sang Min Park

**Affiliations:** 1 Department of Biomedical Sciences, Seoul National University Graduate School, Seoul, Republic of Korea; 2 Department of Preventive Dentistry and Public Oral Health, School of Dentistry and Dental Research Institute, Seoul National University, Seoul, Republic of Korea; 3 Department of Dentistry, School of Dentistry and Dental Research Institute, Seoul National University, Seoul, Republic of Korea; 4 Department of Family Medicine, College of Medicine, Seoul National University, Seoul, Republic of Korea

**Keywords:** Epidemiology, Outcomes research

## Abstract

We aimed to evaluate the risk of coronary heart disease (CHD) according to dental caries status in middle-aged patients using a population-based cohort database containing medical/dental claims, health examination, and death records in the Republic of Korea. A total of 234,597 patients were identified in the database who were without history of cardiovascular disease, including 104,638 patients without dental caries, 41,696 with incipient/moderate stage dental caries, and 88,262 advanced/severe dental caries. We used Cox proportional hazards model adjusted for sociodemographic, lifestyle, and medical characteristics to compute hazard ratio (HR) and 95% confidence intervals (95% CI) for CHD according to severity of dental caries. During 1,491,190 person-years of follow-up, there were a total of 6,015 CHD events. After adjustment for potential confounders, patients in the highest quartile of outpatient visits for advanced/severe stage dental caries was associated with an increase in CHD risk (HR = 1.13; 95% CI: 1.04–1.22) as compared with patients without dental caries. When the analysis was restricted to the patients with advanced/severe dental caries, dose-response relationship between number of outpatient visits for dental caries and risk of CHD was observed (*P*_*trend*_: <0.001). Prevention and control of dental caries might be worth promoting in clinical practice to prevent CHD.

## Introduction

Several risk factors have been established for coronary heart disease (CHD) by the studies that examined a wide range of attributes such as genetic marker, lifestyle, and health status^1–4^. Among the lifestyle-related factors, poor dental health or oral hygiene have been reported to be associated with an increased risk of CHD by several observational studies^5–7^. A recent meta-analysis of 15 cohort studies including 230,406 participants found a statistically significant association between periodontal disease and CHD, but severity or chronicity of periodontal disease in these studies might vary according to the definition used in each study^6^. While a large body of evidence indicate a well-established relationship of periodontal disease and CHD, there are not enough studies that explored the association of dental caries and CHD.

Few studies have evaluated association of dental caries with atherosclerosis and cardiometabolic risk factors with a small number of patients^8,9^. However, these studies are susceptible to selection bias and lacks generalizability. Also, these studies did not examine the association between dental caries status and CHD in their population sample. Since dental caries and CHD^10,11^ are common, and are of a public health concern, examining their association  could update the evidence on the relation of dental health and heart disease.

Despite the efforts to improve oral hygiene through teeth brushing and using fluoride, dental caries still remains as one of the most common dental diseases worldwide^12^. While the extent to which dental caries affects CHD is unclear due to insufficient evidence, patients with different severity, activity, and chronicity of dental caries may need additional clinical attention for heart disease. Therefore, we used a large representative sample of medical/dental claims data linked to health examination records and death registry to evaluate the association between severity of dental caries and CHD in middle aged men and women.

## Results

### Characteristics of the study population

The total population of patients with and without dental caries identified from the NHIS-HEALS database were 234,597. Of these patients, 104,638 (44.6%) were without dental caries, and 129,959 (55.4%) were with dental caries with at least two outpatient visits. Among the patients with dental caries, 41,969 (32.3%) were at incipient/moderate stage dental caries, and 88,263 (67.7%) were at advanced/severe dental caries (Table 1).

**Table 1:** Baseline sociodemographic, lifestyle, and medical characteristics of the patients in the National Health Insurance Service-Health Screening Cohort (NHIS-HEALS) according to dental caries status.

	Total (*N* = *234,597*)	Without Dental Caries (*N* = *104,638*)	With Dental Caries^a^	
			Incipient/Moderate Stage^b^ (*N* = *41,696*)	Advanced/Severe Stage^c^ (*N* = *88,263*)
Age, mean (SD)	54.3 (8.8)	54.4 (9.1)	52.9 (8.3)	55.0 (8.6)
Men	132,119 (56.4)	59,657 (57.1)	21,749 (52.2)	50,713 (57.5)
**Residential area**				
Capital	37,813 (16.1)	15,899 (15.2)	7,352 (17.6)	14,562 (16.5)
Metropolitan	108,645 (46.4)	47,744 (45.7)	19,901 (47.8)	41,000 (46.5)
City/Town	88,139 (37.5)	40,995 (39.1)	14,443 (34.6)	32,701 (37.0)
**Insurance premium** ^**d**^				
1Q	33,169 (14.2)	15,351 (14.7)	5,248 (12.6)	12,570 (14.3)
2Q	48,721 (20.8)	22,672 (21.7)	7,648 (18.4)	18,401 (20.9)
3Q	66,268 (28.3)	29,735 (28.4)	11,292 (27.1)	25,241 (28.6)
4Q	86,439 (36.7)	36,880 (35.2)	17,508 (41.9)	32,051 (36.2)
**Cigarette smoking**				
Non-smoker	160,239 (68.3)	70,561 (67.4)	30,105 (72.2)	59,573 (67.5)
Past smoker	22,641 (9.7)	9,998 (9.6)	4,009 (9.6)	8,634 (9.8)
Current smoker	51,717 (22.0)	24,079 (23.0)	7,582 (18.2)	20,056 (22.7)
**Alcohol consumption**				
Barely any consumption	132,737 (56.6)	57,898 (55.3)	24,309 (58.3)	50,530 (57.3)
2–3 times/month	36,843 (15.7)	16,439 (15.7)	6,780 (16.3)	13,624 (15.4)
1–2 times/week	40,663 (17.3)	18,712 (17.9)	6,946 (16.7)	15,005 (17.0)
3–4 times/week	15,627 (6.7)	7,344 (7.0)	2,483 (6.0)	5,800 (6.6)
Almost everyday	8,727 (3.7)	4,245 (4.1)	1,179 (2.7)	3,304 (3.7)
**Physical activity**				
None or up to 2 times/week	116,730 (49.8)	53,939 (51.6)	19,131 (45.9)	43,660 (49.5)
3–4 times/week	63,800 (27.2)	27,821 (26.6)	12,076 (29.0)	23,903 (27.1)
5–6 times/week	29,128 (12.4)	12,357 (11.8)	5,909 (14.2)	10,862 (12.3)
Almost everyday	24,939 (10.6)	10,521 (10.0)	4,580 (10.9)	9,838 (11.1)
Fasting serum glucose, mg/dL, mean (SD)	97.3 (28.0)	97.6 (28.4)	95.8 (25.6)	97.8 (28.6)
Total cholesterol, mg/dL, mean (SD)	198.3 (36.6)	198.4 (36.9)	197.9 (35.9)	198.6 (36.6)
Body Mass Index, kg/m^2^, mean (SD)	23.9 (2.86)	23.9 (2.89)	23.8 (2.79)	23.9 (2.85)
**Blood pressure, mmHg, mean(SD)**				
SBP	125.7 (16.9)	126.4 (17.1)	124.1 (16.5)	125.7 (16.8)
DBP	78.8 (11.1)	79.2 (11.1)	77.9 (10.9)	78.7 (11.0)
**Charlson comorbidity index**				
0	85,741 (36.9)	39,658 (38.3)	15,212 (36.8)	30,871 (35.3)
1	78,581 (33.8)	34,727 (33.6)	14,292 (34.6)	29,562 (33.8)
2	41,203 (17.7)	17,682 (17.1)	7,289 (17.6)	16,232 (18.6)
≥3	29,072 (11.6)	12,571 (11.0)	4,903 (11.0)	11,598 (12.3)
Family history of heart disease	4,776 (2.2)	2,056 (2.1)	970 (2.5)	1,750 (2.2)

Data above are presented as n(%) unless otherwise noted.

^a^Includes ICD-10 codes for dental caries with at least 2 outpatient visits.

^b^Includes ICD-10 codes for dental caries limited to enamel (K02.0), dental caries of dentin (K02.1), dental caries of cementum, arrested dental caries (K02.3), other dental caries (K02.8), and unspecified dental caries (K02.9) with at least 2 outpatient visits.

^c^Includes ICD-10 codes for irreversible pulpitis (K04.0), necrosis of pulp (K04.1), and periapical abscess with sinus (K04.6) with at least 2 outpatient visits.

^d^Proxy for income status in the NHIS-HEALS.

### Cox proportional hazards model for dental caries and CHD association

Of the study cohort included in the primary analyses, there were 6,015 CHD events during 1,491,190 person-years of follow-up that lasted from January 1, 2006 to December 31, 2013. Across the quartiles of outpatient visits for advanced/severe dental caries, median (interquartile range, IQR) for the number of visits for the first, second, third, and fourth quartiles were 7 (5 to 9), 13 (12 to 15), 21 (19 to 24), and 40 (32 to 55), respectively. Compared with the patients without dental caries, the risk estimate for CHD was the highest among the patients with the most frequent outpatient visits (Quartile 4) for advanced/severe stage dental caries was (HR = 1.13; 95% CI: 1.04–1.22) after adjusting for potential confounders (Table 2).

**Table 2:** Hazard ratios (and 95% confidence intervals) for coronary heart disease in patients with advanced/severe stage dental caries as compared to those without dental caries in the National Health Insurance Service-Health Screening Cohort (NHIS-HEALS).

	Without Dental Caries (*N* = *104,638*)	Advanced/Severe Stage Dental Caries^a^				*P for trend*
		Quartile 1 (*N* = *25,212*)	Quartile 2 (*N* = *19,395*)	Quartile 3 (*N* = *21,605*)	Quartile 4 (*N* = *22,051*)	
**Outpatient visits**						
Median (IQR)		7 (5 to 9)	13 (12 to 15)	21 (19 to 24)	40 (32 to 55)	
**Coronary Heart Disease** ^b^						
No. of Events	3,152	659	595	741	868	
Person-Years	808,004	195,804	150,251	167,268	169,863	
Multivariable Model 1	1 (reference)	0.85 (0.78–0.93)^***^	0.99 (0.90–1.08)	1.09 (1.00–1.18)^*^	1.21 (1.12–1.31)^***^	<0.001
Multivariable Model 2	1 (reference)	0.84 (0.78–0.92)^***^	0.97 (0.89–1.06)	1.06 (0.98–1.15)	1.16 (1.07–1.25)^***^	0.001
Multivariable Model 3	1 (reference)	0.87 (0.80–0.95)^***^	0.98 (0.90–1.08)	1.06 (0.97–1.15)	1.13 (1.04–1.22)^**^	0.005

^a^Includes ICD-10 codes for irreversible pulpitis (K04.0), necrosis of pulp (K04.1), and periapical abscess with sinus (K04.6) with at least 2 outpatient visits.

^b^Includes ICD-10 codes for coronary heart disease (I20–I25).

NOTE: Models represent multivariable Cox regression models.

Model 1 adjusted for age, sex, insurance premium, residential area.

Model 2 adjusted for variables included in Model 1 and year of diagnosis, physical activity, alcohol consumption, cigarette smoking, total cholesterol level, fasting serum glucose level, systolic blood pressure, body mass index.

Model 3 adjusted for variable included in Model 2 and family history of heart disease, and Charlson Comorbidity Index.

Abbreviation: IQR, interquartile range; HR, hazard ratio; CI, confidence intervals; ICD, international classification of diseases

*p < 0.05, **p < 0.01, ***p < 0.001.

### Secondary and subgroup analyses

When the analysis was restricted to the patients with advanced/severe stage dental caries, the risk of CHD in the top quartile (Quartile 4) versus the bottom quartile (Quartile 1) was 1.26 (95% CI: 1.07 1.34) in a dose-response manner (Ptrend <0.001). Patients in the second (HR = 1.12; 95% CI: 1.00–1.26) and third (HR = 1.20: 95% CI: 1.07–1.34) quartiles of outpatient visits for advanced/severe stage dental caries were almost at higher risk for CHD as compared with the patients in the bottom quartile (Table 3).

**Table 3:** Dose-response relationship between advanced/severe stage dental caries and coronary heart disease in the National Health Insurance Service-Health Screening Cohort (NHIS-HEALS).

	Advanced/Severe Stage Dental Caries^a^				*P for trend*
	Quartile 1 *(N* = *25,212)*	Quartile 2 (*N* = *19,395*)	Quartile 3 (*N* = *21,605*)	Quartile 4 (*N* = *22,051*)	
**Outpatient visits**					
Median (IQR)	7 (5 to 9)	13 (12 to 15)	21 (19 to 24)	40 (32 to 55)	
**Coronary Heart Disease** ^**b**^					
No. of Events	659	595	741	868	
Person-Years	195,804	150,251	167,268	169,863	
HR (95% CI), Model 1	1 (reference)	1.16 (1.04–1.29)*	1.28 (1.15–1.42)^***^	1.43 (1.29–1.58)^***^	<0.001
HR (95% CI), Model 2	1 (reference)	1.14 (1.02–1.28)*	1.24 (1.11–1.38)^***^	1.34 (1.21–1.50)^***^	<0.001
HR (95% CI), Model 3	1 (reference)	1.12 (1.00–1.26)	1.20 (1.07–1.34)^**^	1.26 (1.13–1.41)^***^	<0.001

^a^Includes ICD-10 codes for irreversible pulpitis (K04.0), necrosis of pulp (K04.1), and periapical abscess with sinus (K04.6) with at least 2 outpatient visits.

^b^Includes ICD-10 codes for coronary heart disease (I20–I25)

NOTE: Models represent multivariable Cox regression models.

Model 1 adjusted for age, sex, insurance premium, residential area.

Model 2 adjusted for variables included in Model 1 and year of diagnosis, physical activity, alcohol consumption, cigarette smoking, total cholesterol level, fasting serum glucose level, systolic blood pressure, body mass index.

Model 3 adjusted for variable included in Model 2 and family history of heart disease, and Charlson Comorbidity Index.

Abbreviation: IQR, interquartile range; HR, hazard ratio; CI, confidence intervals.

*p < 0.05, **p < 0.01, ***p < 0.001.

We stratified the patients with advanced/severe dental caries by quintiles of outpatient visits for advanced/severe stage dental caries to examine the association of severity of dental caries with risk of CHD, similar associations were observed as compared to the primary analyses (eTables 1 and 2 in the Supplement). When the quartiles of outpatient visit for advanced/severe stage dental caries were re-evaluated by dividing the number of outpatient visits by the number of days between the first diagnosis and the index date, the results were consistent to the analysis that did not take the time of diagnosis into account (eTable 3 in the Supplement). Categorizing the patients by progression of dental caries (incipient/moderate and advanced/severe stage) without using the number of outpatient visits (a proxy for severity) showed that there was no significant association between the presence of dental caries and CHD. Additional cohort analysis matched for age, sex, insurance premium, residential area, and year of diagnosis for dental caries generated similar results (eTables 4 and 5 in the Supplement).

## Discussion

This nationally representative cohort study provides an overview of the association between severity of dental caries and risk of CHD among middle-aged men and women in the Republic of Korea. Our findings demonstrate that advanced/severe stage dental caries was significantly associated with an increase in CHD risk among those with the highest outpatient visits for dental caries as compared with those without dental caries and lowest outpatient visits. Our findings highlight the public health need to control dental caries, along with other well-established risk factors, for prevention of CHD in the middle-aged population.

A few studies have examined the association of dental caries with atherosclerosis and cardiometabolic risk factors. Glodny *et al*. analyzed computed tomography data of 293 patients in Brazil to examine the link between the occurrence of dental caries and atherosclerosis and found a positive relationship of dental caries and atherosclerosis^13^. In this study, dental caries was assessed by the number of decayed surfaces of patients’ teeth and evaluated atherosclerosis with coronary calcium score. In addition, a case-control study by Kelishadi *et al*. reported that dental caries was significantly related to cardiometabolic risk factors (e.g. lipid profile, body mass index, and waist circumference) in adolescents^9^. While these studies provide evidence on dental caries and cardiovascular disease risk factors, none have evaluated the direct relationship of dental caries with risk of CHD. Since the study by Kelishadi *et al*. were conducted in a small sample of adolescent population, observing CHD events may not be reasonable in the restricted time frame. Therefore, further studies are necessary to establish a cause-and-effect relationship between dental caries and well-known cardiometabolic risk factors.

The patients with advanced/severe stage dental caries were those who received endodontic treatment to cease pulpal and periapical infection or prevent recurrent infection. Endodontic treatment involves the removal of inflamed or infected pulpal tissues and bacteria from the root canal system, mechanical and chemical cleaning and shaping, followed by obturation of the decontaminated root canal system, thus often requiring multiple visits. The number of treatment visits increases with an increase in either endodontic treatment difficulty or the number of infected teeth. As the pulpal and periapical infection is an inflammatory disease of bacterial etiology, the number of bacterial count would be relatively greater among the patients with advanced/severe dental caries in the fourth quartile than that among those who visited fewer for endodontic treatment. Despite continuous controversy, numerous studies have reported an association between periapical infection and an increase risk of CHD^14–17^. Bacteremia can occur when the bacteria are directed introduced to the bloodstream by inadvertent instrumentation during endodontic treatment, especially when the instrumentation was performed beyond the confines of root canal space^18^. In addition, the level of inflammatory mediators elevates in patients with apical periodontitis^19,20^, which occurs subsequent to dental caries after pulpal necrosis. It could be speculated that bacteria involved in the root canal system may be a contributing factor of increased risk of CHD. Therefore, the incidence of CHD increased significantly as the number of dental visits for the treatment of advanced/severe stage dental caries increased, particularly in the fourth quartile.

However, the patients with advanced/severe dental caries in the first quartile exhibited higher incidence of CHD than those without dental caries. A possible explanation for the reversal pattern, although not very significant, could be derived from the limitation of the NHIS-HEALS database, since few patients who actually had dental caries were likely to be grouped into patients without dental caries until dental claims are lodged after receiving corresponding dental treatment; some of them may have put off visiting the dentist due to expensive treatment cost, which is regarded as a major barrier to receiving dental care, because both endodontic and prosthetic treatment are typically performed in order to save the tooth with advanced/severe dental caries

The underlying mechanism on the association of dental caries and CHD remains unclear. Severe dental caries may adversely cause chronic inflammatory response in the endothelial coronary cells through bacterial invasion from decayed teeth. Also, severe dental caries can induce atherosclerosis and exacerbate cardiometabolic risk factors that contribute to development of CHD. If these biological mechanisms can be responsible for the association between dental caries and CHD, better designed studies with more controlled factors are necessary to confirm such association.

There are several limitations in our study. First, when assessing severity of dental caries with number of outpatient visits based on dental claims data of the NHIS-HEALS database, we were not able to take number of teeth decayed or treated into account. While the number of teeth with dental caries is important when assessing dental health status, such information was not available in the database. Second, we were unable to show that the patients with severe dental caries were infected with micro-organisms invading endothelial coronary cells and causing inflammatory response because such data could not be found due to the nature of registry-based database. Third, our study consisted of middle-aged men and women who were enrolled in the NHIS system in the Republic of Korea, and therefore has limited generalizability to other age groups and nationalities.

One notable strength of our study is that we were able to conduct a large cohort study with a nationally representative sample linking health and dental claims database to health examination and death registry. We were able to collect various confounders such as lifestyle and co-morbidities from the linked database that were used in the analyses. Also, we were able to minimize the patients who were lost to follow-up by using registry-based dataset. Furthermore, we applied a relatively strict criteria for the CHD event definition to minimize misclassification.

In conclusion, better management of oral hygiene and dental health to prevent progression of dental caries might also lead to reduced CHD risk in the middle-aged population. As previous studies have established a wide range of risk factors for CHD, clinicians such as cardiologists may benefit from considering progression of dental caries as an additional risk factor when assessing CHD risk for patients. While these findings inform that the patients with advanced/severe dental caries may need additional clinical attention for CHD, further studies are necessary to confirm this association and underlying mechanisms prior to developing intervention strategies for improving dental health to prevent CHD in clinical practice.

## Methods

The data for this study were obtained from the National Health Insurance Service-Health Screening Cohort (NHIS-HEALS) database linked to death registry in the Republic of Korea. The NHIS-HEALS is a nationally representative cohort that enrolled insurees of the NHIS aged between 40 and 79 in 2002 and followed through 2013. Individuals enrolled in the NHIS-HEALS were randomly selected from the participants who underwent national health screening program between January 1, 2002 and December 31, 2003 with a 10% sampling rate. The NHIS-HEALS has been used for a wide range of research areas, especially in the field of healthcare policy and epidemiology^21–23^. Further details of the database is available elsewhere^24^. Institutional Review Board (IRB) at the Seoul National University Hospital, which is in compliance with the Declaration of Helsinki, approved this study (IRB Number:1801-019-912). Because the data were routinely collected and anonymized in the NHIS system, we were waived from receiving informed consent from the patients.

**Figure 1:**
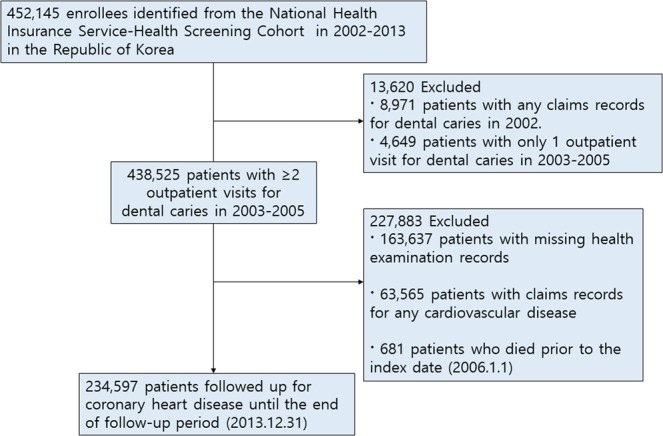
Flow chart of study population from the National Health Insurance Service-Health Screening Cohort database in the Republic of Korea.

We included 452,145 enrollees identified from the NHIS-HEALS database between January 1, 2002, and December 31, 2013. Of the enrollees, we excluded 8,971 patients with any claims records for dental caries in 2002 and 4,649 patients with only one outpatient visit for dental caries from 2003 to 2005 to identify newly diagnosed cases of dental caries in the cohort. Among 438,525 patients with at least two outpatient visits for dental caries from 2003 to 2005, we further excluded 227,883 patients with missing health examination records, medical claims records for any cardiovascular disease, and died before the index date. The final cohort used for this study included 234,597 patients who were followed up for CHD from January 1, 2006 to December 31, 2013 (Fig. 1). Based on the International Classification of Diseases, Tenth revision (ICD-10), we assessed severity of dental caries as follows: patients with claims records with at least two outpatient visits for dental caries limited to enamel (ICD-10 code: K02.0), dental caries of dentin (ICD-10 code: K02.1), dental carries of cementum, arrested dental caries (ICD-10 code: K02.3), other dental caries (ICD-10 code: K02.8), and unspecified dental caries (ICD-10 code: K02.9) were classified as incipient/moderate dental caries, and those with irreversible pulpitis (ICD-10 code: K04.0), necrosis of pulp (ICD-10 code: K04.1), and periapical abscess with sinus (ICD-10 code: K04.6) were classified as advanced/severe stage dental caries. Patients without dental caries were defined as those without claims records for any stage of dental caries.

The primary outcome of this study was CHD. We determined the CHD events based on the medical claims records and admission data in the NHIS-HEALS. To minimize the cases that did not turn out to be the actual CHD events, we only included claims records for CHD (ICD-10 codes: I20–I25) with at least two days of hospital admission for the outcome of this study. Therefore, outpatient records or hospital discharge within one day were excluded when assessing the CHD events in the database. The validity of this identification method for CHD and other cardiovascular disease is available in previous studies^23,25,26^.

Sociodemographic characteristics (age, sex, residential area, and insurance premium), lifestyle factors (cigarette smoking, alcohol consumption, and physical activity) and health status (fasting serum glucose, total cholesterol, blood pressure, body mass index, and family history of heart disease), and medical history (Charlson Comorbidity Index) of the patients were collected from the health insurance eligibility, national health screening, and medical claims database in the NHIS-HEALS, respectively. Charlson Comorbidity Index was calculated based on the medical claims records before the follow-up period to assess the general range of comorbid conditions among the study population. Baseline characteristics of the total study population, patients without dental caries, patients with incipient/moderate and advanced/severe dental caries were reported with number (percentage) for categorical variables and mean (standard deviation) for continuous variables.

In this study, the patients were censored at the first event of CHD, death due to CHD or any other causes, or end of follow-up from January 1, 2006 to December 31, 2013, whichever occurred first. We used Cox proportional hazards model to examine the association between severity of dental caries and CHD, and estimated the risk of CHD with adjusted hazard ratio (HR) and 95% confidence intervals (95% CI) across the different categories of dental caries status. The following variables were used to develop models for the analyses: Model 1 (age, sex, insurance premium, and residential area), Model 2 (variables included in Model 1 and year of dental caries diagnosis, physical activity, alcohol consumption, cigarette smoking, total cholesterol, fasting serum glucose, systolic blood pressure, body mass index), and Model 3 (family history of heart disease and Charlson Comorbidity Index in addition to the variables included in Model 2).

In primary analyses, we computed HRs and 95% CIs for CHD in patients with advanced/severe stage dental caries stratified by quartiles of outpatient visits for dental caries compared with patients without dental caries. To further examine the dose-response relationship between severity of dental caries and CHD, the analytic sample was limited to those with advanced/severe stage dental caries and the risk of CHD was calculated for the patients in the second, third, and fourth quartile of outpatient visits for dental caries compared with the patients in the first quartile. In secondary analyses, we conducted the same analysis with the patients grouped by quintiles of dental caries. To reduce the selection bias due to the time of diagnosis for dental caries, we grouped the patients into quartiles by number of outpatient visits for advanced/severe stage dental caries divided by the number of days between the first diagnosis and index date. Also, we assessed the risk of CHD in patients with any stage of dental caries (incipient/moderate and advanced/severe stage combined), incipient/moderate stage, and advanced/severe stage compared with patients without dental caries with multivariable Cox regression Model 3. In addition, we created a 1:1 matched cohort (matched for age, sex, insurance premium, and year of diagnosis for dental carries) for patients with and without dental caries to examine the association of dental caries with CHD. We used SAS 9.4 (SAS Institute, Cary, NC, USA) and STATA 14.0 (StataCorp LP, College Station, TX, USA) for data collection and statistical analyses for this study.

## Supplementary information

Supplements
