# Supplementary material for: Severity of dental caries and risk of coronary heart disease in middle-aged men and women: a population-based cohort study of Korean adults, 2002–2013
Source: Sci Rep. 2019 Jul 19;9:10491. doi: 10.1038/s41598-019-47029-3 (PMC6642137; doi:10.1038/s41598-019-47029-3)
Supplement: Supplementary file 1 — Supplements [file 41598_2019_47029_MOESM1_ESM.docx]

**[Supplemental Tables]**

**Severity of dental caries and risk of coronary heart disease in middle-aged men and women:**

**a population-based cohort study of Korean adults, 2002-2013**

**Running Title:** *Kim et al; Dental caries and coronary heart disease*

Kyuwoong Kim^a^, Seulggie Choi^a^, Jooyoung Chang^a^, Sung Min Kim^a^, Seon Jip Kim^b^,
Ryan Jin-Young Kim^c^, Hyun-Jae Cho^b*^, Sang Min Park^a,d*^

^a^Department of Biomedical Sciences, Seoul National University Graduate School, Seoul, Republic of Korea

^b^Department of Preventive Dentistry and Public Oral Health, School of Dentistry and Dental Research Institute, Seoul National University, Republic of Korea

^c^Department of Dentistry, School of Dentistry and Dental Research Institute, Seoul National University, Republic of Korea

^d^Department of Family Medicine, College of Medicine, Seoul National University, Seoul, Republic of Korea

**Supplemental Table 1.** Multivariable-adjusted hazard ratios (and 95% confidence intervals) for coronary heart disease in patients with advanced/severe stage dental caries as compared to those without dental caries in the National Health Insurance Service-Health Screening Cohort (NHIS-HEALS).

|  |  | Advanced/Severe Stage Dental Caries^a^ | | | | |  |
| --- | --- | --- | --- | --- | --- | --- | --- |
|  | Without Dental Caries  (N=104,638) | Quintile 1  *(N=17,908)* | Quintile 2  (*N*=17,767) | Quintile 3  (*N*=18,677) | Quintile 4  (*N*=16,469) | Quintile 5  (N=17,442) | *P for trend* |
| Outpatient Visits |  |  |  |  |  |  |  |
| Median (IQR) |  | 6 (4 to 7) | 11 (10 to 12) | 17 (15 to 18) | 25 (23 to 28) | 45 (37 to 61) |  |
| Coronary Heart Disease^b^ |  |  |  |  |  |  |  |
| No. of Events | 3,152 | 449 | 532 | 592 | 593 | 697 |  |
| Person-Years | 808,004 | 139,137 | 137,724 | 144,594 | 127,343 | 134,387 |  |
| Multivariable Model 1 | 1 (reference) | 0.82  (0.74-0.91)^***^ | 0.97  (0.88-1.06) | 1.01  (0.93-1.11) | 1.14  (1.04-1.25)^**^ | 1.22  (1.12-1.32)^**^ | <0.001 |
| Multivariable Model 2 | 1 (reference) | 0.81  (0.74-0.90)^***^ | 0.96  (0.87-1.05) | 0.99  (0.91-1.08) | 1.11  (1.01-1.21)^*^ | 1.16  (1.05-1.26)^**^ | 0.001 |
| Multivariable Model 3 | 1 (reference) | 0.84  (0.75-0.93)^**^ | 0.97  (0.88-1.07) | 1.00  (0.91-1.10) | 1.10  (1.00-1.20) | 1.13  (1.04-1.24)^**^ | 0.003 |

^a^Includes ICD-10 codes for irreversible pulpitis (K04.0), necrosis of pulp (K04.1), and periapical abscess with sinus (K04.6) with at least 2 outpatient visits

^b^Includes ICD-10 codes for coronary heart disease (I20-I25)

NOTE: Models represent multivariable Cox regression models.

Model 1 adjusted for age, sex, insurance premium, residential area

Model 2 adjusted for variables included in Model 1 and year of diagnosis, physical activity, alcohol consumption, cigarette smoking, total cholesterol level, fasting serum glucose level, systolic blood pressure, body mass index,

Model 3 adjusted for variable included in Model 2 and family history of heart disease, and Charlson Comorbidity Index.

Abbreviation: IQR, interquartile range; HR, hazard ratio; CI, confidence intervals; ICD, international classification of diseases

*p<0.05, **p<0.01, ***p<0.001

**Supplemental Table 2.** Association between advanced/severe stage dental caries and coronary heart disease in the National Health Insurance Service-Health Screening Cohort (NHIS-HEALS).

|  | Advanced/Severe Stage of Dental Caries^a^ | | | | |  |
| --- | --- | --- | --- | --- | --- | --- |
|  | Quintile 1  *(N=17,908)* | Quintile 2  (*N*=17,767) | Quintile 3  (*N*=18,677) | Quintile 4  (*N*=16,469) | Quintile 5  (N=17,442) | *P for trend* |
| Outpatient Visits |  |  |  |  |  |  |
| Median (IQR) | 6 (4 to 7) | 11 (10 to 12) | 17 (15 to 18) | 25 (23 to 28) | 45 (37 to 61) |  |
| Coronary Heart Disease^b^ |  |  |  |  |  |  |
| No. of Events | 449 | 532 | 592 | 593 | 697 |  |
| Person-Years | 139,137 | 137,724 | 144,594 | 127,343 | 134,387 |  |
| HR (95% CI), Model 1 | 1  (reference) | 1.18  (1.04-1.34)^**^ | 1.24  (1.09-1.40)^**^ | 1.40  (1.23-1.58)^***^ | 1.50  (1.33-1.69)^***^ | <0.001 |
| HR (95% CI), Model 2 | 1  (reference) | 1.17  (1.03-1.33)^*^ | 1.20  (1.06-1.36)^**^ | 1.34  (1.18-1.52)^***^ | 1.40  (1.23-1.58)^***^ | <0.001 |
| HR (95% CI), Model 3 | 1  (reference) | 1.16  (1.01-1.32)^*^ | 1.18  (1.04-1.35)^**^ | 1.29  (1.13-1.47)^***^ | 1.32  (1.16-1.50)^***^ | <0.001 |

^a^Includes ICD-10 codes for irreversible pulpitis (K04.0), necrosis of pulp (K04.1), and periapical abscess with sinus (K04.6) with at least 2 outpatient visits

^b^Includes ICD-10 codes for coronary heart disease (I20-I25)

NOTE: Models represent multivariable Cox regression models.

Model 1 adjusted for age, sex, insurance premium, residential area

Model 2 adjusted for variables included in Model 1 and year of diagnosis, physical activity, alcohol consumption, cigarette smoking, total cholesterol level, fasting serum glucose level, systolic blood pressure, body mass index,

Model 3 adjusted for variable included in Model 2 and family history of heart disease, and Charlson Comorbidity Index.

Abbreviation: IQR, interquartile range; HR, hazard ratio; CI, confidence intervals; ICD, international classification of diseases

*p<0.05, **p<0.01, ***p<0.001

**Supplemental Table 3.** Analyses of the association between advanced/severe stage dental caries and coronary heart disease with number of diagnoses and time until the index date in the National Health Insurance Service-Health Screening Cohort (NHIS-HEALS).

|  |  | Advanced/Severe Stage Dental Caries^a^ | | | |  |
| --- | --- | --- | --- | --- | --- | --- |
|  | Without Dental Caries  (N=104,638) | Quartile 1  (N=22,069) | Quartile 2  (N=22,066) | Quartile 3  (N=22,078) | Quartile 4  (N=22,050) | *P for trend* |
| Coronary Heart Disease^b^ |  |  |  |  |  |  |
| No. of Events | 3,152 | 590 | 685 | 743 | 845 |  |
| Person-Years | 808,004 | 171,178 | 171,016 | 170,939 | 170,056 |  |
| HR (95% CI), Model 1 | 1 (reference) | 0.87  (0.80-0.95)^**^ | 1.00  (0.91-1.08) | 1.08  (0.99-1.17) | 1.19  (1.10-1.28)^***^ | <0.001 |
| HR (95% CI), Model 2 | 1 (reference) | 0.87  (0.79-0.95)^**^ | 0.97  (0.89-1.06) | 1.05  (0.96-1.13) | 1.13  (1.05-1.23)^**^ | <0.001 |
| HR (95% CI), Model 3 | 1 (reference) | 0.90  (0.82-0.99)^*^ | 0.96  (0.88-1.05) | 1.06  (0.97-1.15) | 1.11  (1.02-1.20)^*^ | <0.001 |

^a^Includes ICD-10 codes for irreversible pulpitis (K04.0), necrosis of pulp (K04.1), and periapical abscess with sinus (K04.6) with at least 2 outpatient visits. Patients were grouped into quartiles by the following formula: number of outpatient visits for advanced/severe stage dental caries divided by the number of days between the first diagnosis and the index date.

^b^Includes ICD-10 codes for coronary heart disease (I20-I25)

NOTE: Models represent multivariable Cox regression models.

Model 1 adjusted for age, sex, insurance premium, residential area

Model 2 adjusted for variables included in Model 1 and year of diagnosis, physical activity, alcohol consumption, cigarette smoking, total cholesterol level, fasting serum glucose level, systolic blood pressure, body mass index,

Model 3 adjusted for variable included in Model 2 and family history of heart disease, and Charlson Comorbidity Index.

Abbreviation: HR, hazard ratio; CI, confidence intervals; ICD, international classification of diseases

*p<0.05, **p<0.01, ***p<0.001

**Supplemental Table 4.** Multivariable-adjusted hazard ratios (and 95% confidence intervals) for coronary heart disease according to dental caries status in the National Health Insurance Service-Health Screening Cohort (NHIS-HEALS)..

|  |  |  | Progression of Dental Caries | |
| --- | --- | --- | --- | --- |
|  | Without  Dental Caries  (*N=104,638*) | With Dental Caries  (*N=129,959*) | Incipient/Moderate  Stage^a^  (*N=41,696*) | Advanced/Severe  Stage^b^  (*N=88,263*) |
| Coronary Heart Disease^c^ |  |  |  |  |
| No. of Events | 3,152 | 3,980 | 1,117 | 2,863 |
| Person-Years | 808,004 | 1,008,210 | 325,022 | 683,188 |
| HR (95% CI), Model 1 | 1 (reference) | 1.01 (0.96-1.05) | 0.97 (0.91-1.04) | 1.02 (0.97-1.07) |
| HR (95% CI), Model 2 | 1 (reference) | 1.02 (0.97-1.07) | 1.01 (0.94-1.07) | 1.02 (0.97-1.08) |
| HR (95% CI), Model 3 | 1 (reference) | 1.02 (0.97-1.07) | 1.01 (0.94-1.08) | 1.02 (0.97-1.07) |

^a^Includes ICD-10 codes for dental caries limited to enamel (K02.0), dental caries of dentin (K02.1), dental caries of cementum, arrested dental caries (K02.3), other dental caries (K02.8), and unspecified dental caries (K02.9) with at least 2 outpatient visits.

^b^Includes ICD-10 codes for irreversible pulpitis (K04.0), necrosis of pulp (K04.1), and periapical abscess with sinus (K04.6) with at least 2 outpatient visits.

^c^Includes ICD-10 codes for coronary heart disease (I20-I25)

NOTE: Models represent multivariable Cox regression models.

Model 1 adjusted for age, sex, insurance premium, residential area

Model 2 adjusted for variables included in Model 1 and year of diagnosis, physical activity, alcohol consumption, cigarette smoking, total cholesterol level, fasting serum glucose level, systolic blood pressure, body mass index,

Model 3 adjusted for variable included in Model 2 and family history of heart disease, and Charlson Comorbidity Index.

Abbreviation: HR, hazard ratio; CI, confidence intervals; ICD, international classification of diseases

*p<0.05, **p<0.01, ***p<0.001

**Supplemental Table 5.** Association between dental caries and coronary heart disease in a matched cohort analysis of the National Health Insurance Service-Health Screening Cohort (NHIS-HEALS).

|  | Without Dental Caries  (*N=104,536*) | With Dental Caries^a^  (*N=104,536*) |
| --- | --- | --- |
| Coronary Heart Disease^b^ |  |  |
| No. of Events | 3,151 | 3,169 |
| Person-Years | 807,193 | 810,576 |
| Unadjusted | 1 (reference) | 1.00 (0.95-1.05) |
| Adjusted for CHD risk factors^c^ | 1 (reference) | 1.01 (0.97-1.07) |
| Adjusted for CHD risk factors and  Comorbidity^d^ | 1 (reference) | 1.00 (0.95-1.05) |
| Adjusted for CHD risk factors,  Comorbidity, and family history^e^ | 1 (reference) | 1.01 (0.96-1.06) |

Propensity score matching for age, sex, insurance premium, residential area, and year of diagnosis for dental caries using greedy nearest neighbor matching (lowest to highest).

^a^Includes both incipient/moderate and advanced/severe stage of dental caries with at least 2 outpatient visits.

^b^Includes ICD-10 codes for coronary heart disease (I20-I25).

^c^Lifestyle factors (physical activity, alcohol consumption, and cigarette smoking) and clinical factors (total cholesterol, fasting serum glucose, blood pressure, body mass index).

^d^Charlson comorbidity index.

^e^Family history of heart disease.
